# Study on Rice Grain Mildewed Region Recognition Based on Microscopic Computer Vision and YOLO-v5 Model

**DOI:** 10.3390/foods11244031

**Published:** 2022-12-14

**Authors:** Ke Sun, Yu-Jie Zhang, Si-Yuan Tong, Meng-Di Tang, Chang-Bao Wang

**Affiliations:** 1 College of Life Sciences, Anhui Normal University, Wuhu 241000, China; 2 College of Food Science and Technology, Nanjing Agricultural University, Nanjing 210095, China

**Keywords:** computer vision, microscopic image, convolutional neural network, mildewed rice grain, rapid detection

## Abstract

This study aims to develop a high-speed and nondestructive mildewed rice grain detection method. First, a set of microscopic images of rice grains contaminated by *Aspergillus niger*, *Penicillium citrinum*, and *Aspergillus cinerea* are acquired to serve as samples, and the mildewed regions are marked. Then, three YOLO-v5 models for identifying regions of rice grain with contamination of *Aspergillus niger*, *Penicillium citrinum*, and *Aspergillus cinerea* in microscopic images are established. Finally, the relationship between the proportion of mildewed regions and the total number of colonies is analyzed. The results show that the proposed YOLO-v5 models achieve accuracy levels of 89.26%, 91.15%, and 90.19% when detecting mildewed regions with contamination of *Aspergillus niger*, *Penicillium citrinum*, and *Aspergillus cinerea* in the microscopic images of the verification set. The proportion of the mildewed region area of rice grain with contamination of *Aspergillus niger*/*Penicillium citrinum*/*Aspergillus cinerea* is logarithmically correlated with the logarithm of the total number of colonies (*TVC*). The corresponding determination coefficients are 0.7466, 0.7587, and 0.8148, respectively. This study provides a reference for future research on high-speed mildewed rice grain detection methods based on MCV technology.

## 1. Introduction

During rice grain storage, the changes in the local temperature and moisture in grain piles can easily lead to the proliferation of molds of the *Aspergillus* and *Penicillium* genera on the grain surface. The mildewed rice grains have low nutritional value and may contain mycotoxin [1], which causes serious food safety problems when such problematic rice products are sold to consumers. Therefore, performing mildew detection on rice grains at different stages, i.e., from harvesting to retailing, is an important measure to ensure the nutrition and safety of rice grains and related food products. At present, the microbial indexes of rice grains are usually determined using the microbial culture method. However, the traditional microbial culture method is costly and time-consuming and therefore cannot meet the requirements of on-the-spot detection at the places of grain purchase/selling. So, a rapid and non-destructive method for evaluating microbial indicators of rice grain is needed.

Spectroscopic and image-based methods have the advantages of low cost, no pre-processing and high speed, and have a high application value in grain quality evaluation.

In the research works regarding the utilization of spectrum and hyper spectrum, Singh et al. (2012) developed a method for detecting fungal infection of wheat ears based on the hyperspectral imaging technology (near-infrared band) [2]; Zhang et al. (2014) acquired the near-infrared spectrum of rice grains contaminated by molds by using a Fourier near-infrared analyzer and established an aflatoxin prediction model [3]; Siripatrawan et al. (2015) devised a method for monitoring the growth of rice molds based on hyperspectral imaging technology [4]; Jayme et al. (2017) developed a method for detecting deoxynivalenol in wheat grains based on hyperspectral imaging technology (wavelength ranging from 528–1785 nm) [5]; Shen et al. (2016, 2018) devised a method for detecting rice mildew caused by brown rice aflatoxin B_1_ and four mold species based on the analysis of near-infrared spectrum [6,7]. Chu et al. (2022) used near-infrared hyperspectral images to identify mildewed corn grains by combining principal component analysis, a continuous projection algorithm and a support vector machine model. The recognition accuracy was close to 100% [8]. In the research works regarding the utilization of computer vision, Wang et al. (2018) developed a method for detecting five mold species on the culture medium based on the computer vision technology [9]; Sun et al. (2016) used the computer vision method and a deep learning model of LeNet5 to detect the contamination of five mold species on rice grains, achieving an accuracy level higher than 90% for all five mold species [10].

Payman et al. (2018) developed an expert system to detect the size, breakage and crack of rice, achieving an accuracy of 96%. In this system, a scanner was used to capture the images of rice and a traditional image processing method was used to extract the features from the images [11]. Chen et al. (2019) used computer vision combined with a support vector machine model to evaluate the breakage and chalkiness of red indica rice, and the accuracy was more than 93% [12]. Shamin et al. (2020) used computer vision combined with a five-layer convolutional neural network (CNN) to classify rice varieties, and the accuracy increased by at least 25% compared with conventional machine learning models [13]. Jeyaraj et al. (2022) developed a real-time scanning system to identify rice varieties. AlexNet was used to classify the images of different varieties of rice, and the classification accuracy was above 98% [14].

Among the quality detection methods of grain described above, the phenotyping of grain, such as size, breakage, and variety of samples, can be accurately detected using computer vision. However, mildew detection in grain has low sensitivity when using traditional spectroscopic and image-based methods. For a mildly mildewed rice grain with a total number of colonies (*TVC*) of 10^5^–10^6^ CFU/g [15], it is difficult to be identified using spectroscopic and image-based methods which identify samples according to the macrophysical and chemical characteristics [10,16]. So, we consider using the microscopic computer technology (MCV), which can capture the microscopic details of samples, to solve this problem.

Microscopic computer vision is a technology that is used to analyze and recognize the features of images acquired by microscopic cameras [17,18]. As compared with the common computer vision technology used to process the macroscopic images, MCV technology is more suitable for identifying the image features associated with the growth of microbacterial colonies on the surface of rice grains. In addition, the latter method achieves higher mildew detection sensitivity and improves the accuracy of detecting samples with mild mildew. However, as the microscopic images contain more information and are more complex than the common images, it is difficult to extract the features from the microscopic images using conventional image processing techniques, such as threshold segmentation and feature parameter extraction. Therefore, microscopic images captured using MCV should be recognized by advanced image recognition methods.

The innovation of image pattern recognition methods promotes the development of computer vision technology [19,20]. The development of image pattern recognition technology has undergone a transformation from conventional machine learning methods to CNN methods [19]. Compared with conventional machine learning models, CNN models automatically extract the image features from the samples and then classify the features for performing accurate recognition. Due to the depth of CNN models, they have strong feature recognition abilities in processing complex images [21,22]. Because the mildewed regions on rice grains are relatively small, it is difficult to identify the mildewed rice grain according to the overall information of the image. So, the mildewed regions on rice grain should be segmented out, and then the mildewed rice grain can be detected with the information of the mildewed regions. Therefore, the basic task is mildewed region segmentation. Due to the complexity of the microscopic image of a rice grain, conventional threshold segmentation methods cannot obtain a good segmentation result, while some of the CNN models, such as region-based CNN (R-CNN), Faster R-CNN, Mask R-CNN, and YOLO (You Look Only Once), can be used to recognize and segment the interested objects in the image [23]. Among these CNN models, YOLO is a deep learning algorithm for fast target detection. It detects, classifies, and locates targets in an image in one go [24]. As compared with R-CNN models, YOLO greatly improves the detection speed while maintaining sufficient accuracy. Consequently, it has been used in various applications, such as vehicle recognition, pedestrian counting, face recognition, and grain recognition [25,26,27,28].

In summary, microscopic computer vision can capture the microscopic image of rice grain, and the YOLO model can be used to identify the small colonies in the microscopic image of rice grain. However, there is much interference information in the microscopic image, including the hull edge, glume protection, and fluff. Therefore, in order to detect mildewed rice grain using the MCV and YOLO models, there are two questions that need to be studied. (1) Whether the YOLO model can identify the mildew regions without interference from other information. (2) Whether the mildew degree of rice grain can be characterized by the mildewed regions identified from the microscopic image.

In order to solve these questions, in this study, a set of microscopic images of single rice grains with different mildew degrees caused by *Aspergillus niger*, *Penicillium citrinum*, and *Aspergillus glaucus* are chosen as the samples, and the regions of bacterial colonies are marked manually. Then, YOLO-v5 models for recognizing bacterial colonies in microscopic images are established and verified. After that, these models are used to estimate the proportion of mildewed regions on the surface of a rice grain (*MAI*) and analyze the relationship between the *MAI* value and the *TVC* value of each rice grain. Finally, a mildewed rice grain detection method based on the MCV and YOLO-v5 models is obtained.

## 2. Methods

### 2.1. Simulated Storage of Rice after Inoculation

*A. niger*, *P. citrinum*, and *A. glaucus* are dominant strains that infect rice grains during storage. Among them, *A. niger* and *P. citrinum* produce ochratoxin and penicillin, which are very toxic, while *Aspergillus aeruginosa* can grow in a relatively dry environment. *A. aeruginosa* is the precursor strain in the process of the dominant strains during storage, replacing the dominant strains in the field [29,30]. In this study, we chose the above three mold strains to perform the experiments. The three mold strains were purchased from Beina Biotechnology (Zhengzhou, China).

The procedures for mold inoculation and simulated storage are as follows:

(1) Select a certain number of clean and mildew-free rice grains (indica rice, purchased from Huainan, Anhui Province, China). Put the sample grains into an oven and bake them at 80 °C for 4 h to kill the original field molds attached to the rice grains. Put the dried sample grains into a set of 90-mm round petri dishes (15 g grains for each petri dish).
(2) Inoculate the three mold strains into the potato glucose agar (PDA) medium separately and activate them at 28 °C for 3 days. Elute the activated colonies with sterile distilled water to prepare spore suspension samples, measure the spore concentrations in the spore suspension samples using the plate counting method, and dilute the spore suspension samples of three molds to 1.5 × 10^4^ CFU/mL.
(3) Take 30 petri dishes containing 15 g of rice grains. Inoculate 1.5 mL of spore suspension to the sample grains in each petri dish (each kind of spore suspension will inoculate 10 Petri dishes). Add 1 mL of sterile water into each petri dish and shake the Petri dish to allow the grains to fully absorb the water. After inoculation, the moisture content of the rice grains will be greater than 20%, thus creating a suitable condition for simulating accelerated mold growth in rice grains in a highly humid environment. Then, place the inoculated sample grains in a constant temperature and humidity incubator and simulate rice storage under the conditions of 28 °C and 90% relative humidity. Take out five grain samples randomly from each petri dish every day to test the degree of mildew. The simulated storage will last 13 days until the grains reach a high mildew degree. During the course of the simulated storage, 1950 sample rice grains with different contamination levels of *A. niger*, *P. citrinum*, and *A. cinerea* are obtained (650 samples for each mold strain).

### 2.2. Acquisition of Rice Grain Microscopic Image

**Figure 1:**
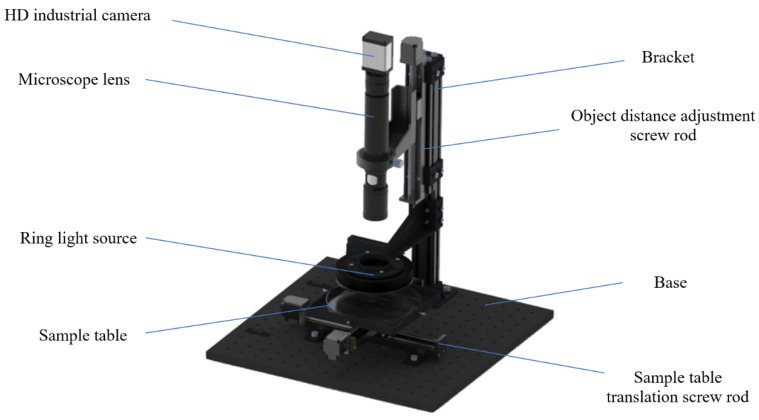
The MCV image acquisition system.

In order to acquire microscopic images of rice grains, we set up an MCV image acquisition system, as shown in Figure 1.

The system is composed of an HD industrial camera, microscope lens, LED ring light source, sample table, bracket, base, object distance adjustment screw rod, and sample table translation screw rod. The HD industrial camera is a Dahua A7A20MU30 color area array industrial camera (Huaray Technology Inc., Hangzhou, China) with a 1.2-inch target surface and a resolution of 4096 × 3000 pixels. The microscope lens is a Lapson high-power lens, which supports 9X optical amplification. The LED ring light source has a power of 10 W and a color temperature of 6500 K. In addition, its light divergence is uniform, and it will not produce a shadow when irradiating the sample grains. The sample table is square, on which petri dishes of different sizes can be placed and fixed. The light source is fixed at the position of 20 mm on the sample table. The height of the microscope lens and industrial camera can be adjusted based on the object distance by using the screw rod controlled by the computer, thus enabling automatic focusing during shooting. Two-dimensional translational movement of the sample table is realized through the action of the translation screw rod controlled by the computer.

**Figure 2:**
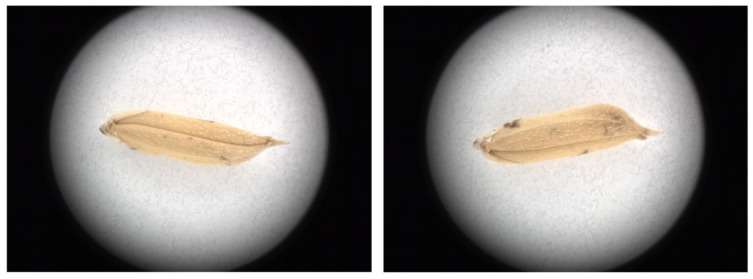
Microscopic images of a rice grain (two sides of the same rice grain).

The procedure for acquiring microscopic images of rice grains is as follows. First, turn on the ring light source. Then, place a single rice grain on the sample table and adjust the position of the sample table until the rice grain is at the center of the camera’s field of vision. Start the automatic adjustment of the object distance to obtain a clear image and acquire the microscopic image of the rice grain. Turn the rice grain over along its central axis and adjust the position of the sample table and the object distance to obtain a clear picture before acquiring the image of the second side of the rice grain. Thus, two microscopic images are acquired for each rice grain (as shown in Figure 2). The image acquisition parameters include an exposure time of 20 ms, a gain of 1 dB, and automatic white balance. The size of the captured images is 4096 × 3000 pixels, and the spatial resolution of a single pixel is about 0.01 mm. The above parameter settings ensure that a whole rice grain is contained in a single image and all the details can be captured clearly. 

### 2.3. Pre-Process of Microscopic Images of Rice Grains

**Figure 3:**
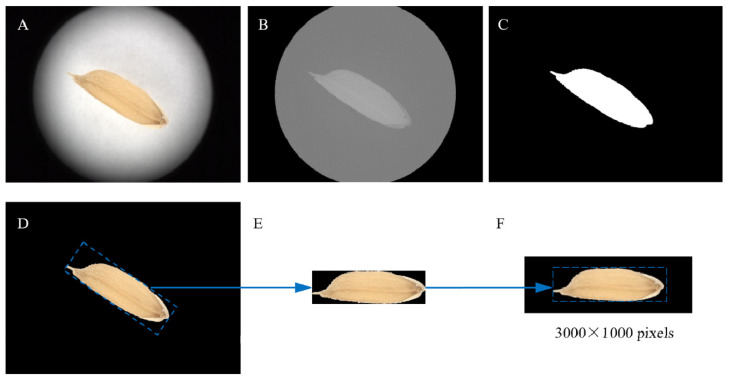
Pre-process of microscopic image of rice grains. (**A**) The original image of rice grain; (**B**) B-component gray image; (**C**) Binary image of the grain; (**D**) Image after the background is removed; (**E**) Grain area obtained through rotation and cropping; (**F**) Grain image obtained after placing the cut-off grain area on a fixed size black image.

Each rice grain image contains a large portion of background, and the orientation of the grain placed on the sample table is not fixed (see Figure 2). These factors create some difficulties for the subsequent analysis. Therefore, it is necessary to perform preprocessing on the acquired microscopic images of rice grains. The preprocessing is performed using MATLAB 2014b (Mathworks Inc., Natick, MA, USA). The steps are as follows: (1) the color space of the original image (Figure 3A) is converted to the lab mode. There is a large difference between the gray-scale values of rice grain and the background in the B-component. Therefore, the B-component gray image (Figure 3B) is extracted for segmenting the rice grain region. Then, the B-component gray image is segmented using the automatic bimodal threshold segmentation method to obtain the binary image of the grain area (Figure 3C). (2) The background of the original image is removed by using the binary image of the grain area as the template. Then, the minimum circumscribed rectangle of the grain in the binary image is calculated, and the angle between the long side of the rectangle and the horizontal direction is calculated. (3) The area within the minimum circumscribed rectangle of the grain is cropped from the original image and is then rotated in the horizontal direction (Figure 3E). Finally, the cropped grain image is placed at the center of a black image of size 3000 × 1000 pixels to obtain the preprocessed image (Figure 3F). After preprocessing, the size of the microscopic image is reduced from 4096 × 3000 pixels to 3000 × 1000 pixels. Finally, all the microscopic images of rice grains are the same size, and the grains in the images have the same orientation. 

### 2.4. Image Marking

**Figure 4:**
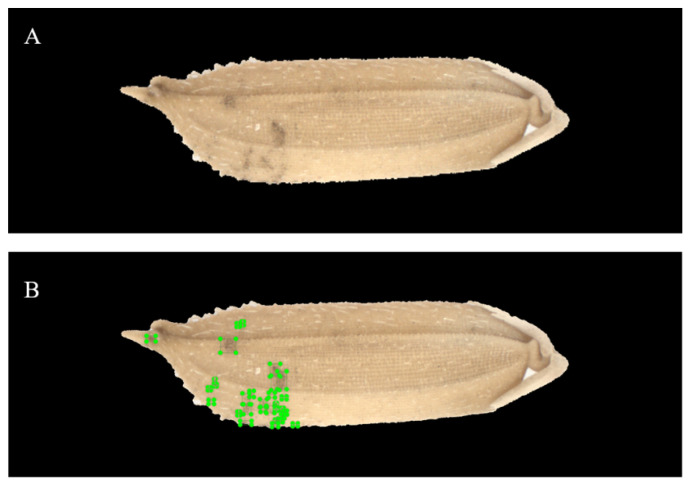
Marking of mildewed regions in rice microscopic images. (**A**) Original image; (**B**) Image with mildewed regions.

The YOLO model is a deep learning method that learns in a supervised manner. It requires manual marking of the mildewed regions in the rice images. As shown in Figure 4, the mildewed regions in all microscopic images are marked using the LabelImg 1.8.6 (Tzutalin, Vancouver, BS, Canada) image marking tool developed using Python. In this study, single category marking is used to mark the mildewed regions without grading the degree of mildew at each mildewed region. In order to obtain more data and more accurate mildewed regions, it is necessary to draw the marking box as close to the outline of the mildewed region as possible, and the size of the bounding boxes does not exceed 200 × 200 pixels.

### 2.5. Model Establishment

**Figure 5:**
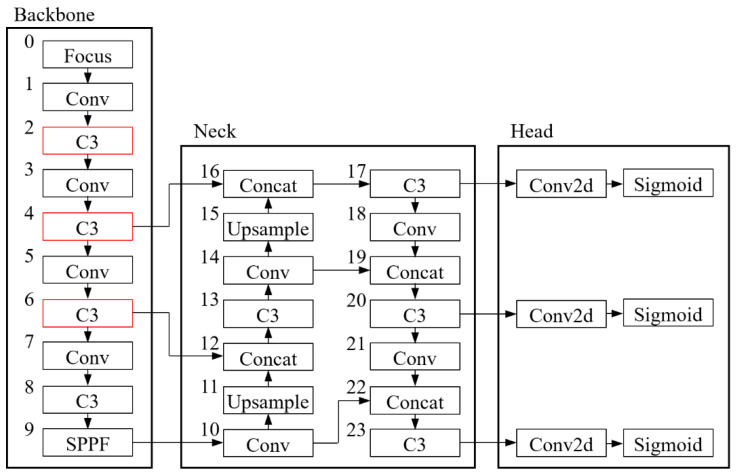
The structure of YOLO-v5 model. The number 0 to 23 is the sequence of the layers in Yolo-v5 model; Three C3 layers to extract the mildewed area from images are highlighted by red boxes.

The fifth generation of the YOLO model (YOLO-v5) is used to establish the model for identifying the mildewed regions on the surface of rice grains. The model architecture is shown in Figure 5. The function of the backbone is to extract features from the image, the function of the neck is to aggregate the image features, and the function of the head is to calculate the model output according to the aggregated image features produced by the neck. The software platform used to establish the model includes Pycharm 2021.3.1 (JetBrains, Prague, Czech Republic). The toolbox used in experiments includes YOLOv5-master, the YOLO-v5 official toolbox based on PyTorch’s deep learning library. The graphics card is an Nvidia RTX 2060 with 6 GB of video memory.

In this study, the three YOLO-v5 models for identifying mildewed regions in the microscopic images of rice grains contaminated by *A. niger*, *P. citrinum*, and *A. cinerea* are established in the following way. 1050 rice grain samples are taken from the prepared rice grains contaminated with *A. niger*, *P. citrinum*, and *A. cinerea* (350 samples for each mold strain). The microscopic images of the samples are acquired (700 images for each mold strain), and the mildewed regions are marked. In the end, 70,497, 62,355, and 50,689 mildewed regions are marked in the microscopic images of rice grains contaminated by *A. niger*, *P. citrinum*, and *A. cinerea*, respectively. The microscopic images of rice grains contaminated by the three mold strains are randomly divided into a training set and a verification set according to the ratio of 6:4 for establishing the models for recognizing mildewed regions of rice grains contaminated by the three mold strains. In order to improve the training speed and reduce memory occupation, we chose the YOLO-v5 s model (a simplified YOLO-v5 model), which has a relatively small number of layers and small number of nodes, for establishing the mildewed region recognition model. The learning rate is set to 0.01. Mosaic enhancement (mosaic), image right-left flipping (Fliplr), and image up-down flipping (Flipud) strategies are used for image data enhancement. The classification model of the COCO dataset is used as the pre-trained model. As many marked mildewed regions are tiny mildew spots (about 20 × 20 pixels), it is difficult for the model to detect such tiny mildew spots if the input resolution of the image is too low. Therefore, the input image size is set to 1280 × 448 pixels. The batch size is set to 4 so as to save the video memory, and the number of training epochs is set to 100 during the training process. In order to reduce the chance of the model misjudging the background and improve the degree of fit between the model output boundary box and the mildewed region, we set both the confidence threshold and intersection-over-union threshold of the model to 0.3. After training is completed, the best model is selected according to the variation in box loss during the training. The method for calculating *box loss* is shown in Equation (1):

(1) $$Box\;loss=1-\frac{A\cap^{\;}B}{A\cup^{\;}B}$$

where *A* ∩ *B* is the number of pixels in the intersection area of the model-predicted boundary box and the manually marked box, and *A* ∪ *B* is the number of pixels in the union area of model-predicted boundary box and the manually marked box.

Then, the confusion matrix of the recognition results yielded by the model in detecting mildewed regions in the image of rice grains contaminated by molds was calculated to evaluate the accuracy of the model’s detection. Finally, the feature images output by the three C3 modules (marked with red boxes in Figure 5) in the second, fourth, and sixth stages of the backbone part of the model were collected to analyze the effectiveness of the model in extracting the features of mildewed regions of rice grains.

### 2.6. Analysis of the Relationship between MAI and TVC of Rice Grain

Nine hundred rice grain samples were taken from the rice grains contaminated by *A. niger*, *P. citrinum*, and *A. cinerea* (300 samples for each mold strain), and the microscopic images of these samples were acquired. After the microscopic images were preprocessed, the proposed model was used to identify the mildewed regions in the images. After the mildewed regions were identified, the *MAI* value of each rice grain was calculated. The calculation method is shown in Equation (2):

(2) $$MAI=\frac{1}{2}\left(\frac{M_{A}}{S_{A}}+\frac{M_{B}}{S_{B}}\right)$$

where, *MAI* is the proportion of mildewed regions. Let the two sides of each rice grain be called side A and side B, then *M*_A_ is the total area of mildewed regions identified in the microscopic image of side A of the rice grain, *M*_B_ is the total area of mildewed regions identified in the microscopic image of side B of the rice grain, *S*_A_ is the total area of side A of the rice grain in the microscopic image, and *S*_B_ is the total area of side B of the rice grain in the microscopic image.

After the microscopic images of the sample rice grains were acquired, the *TVC* of each rice grain was obtained using the colony plate counting method in the following way. First, weigh the mass of the rice grain, and then put it in the test tube. Add 10 mL of sterile, distilled water into the test tube. Then, put the test tube into a shaking table and let the shaking table work for 1 h at a speed of 150 R/min so as to fully elute the mold spores on the grain surface. After the operation of the shaking table is over, dilute the resulting bacterial suspension 10 times, 100 times, and 1000 times to obtain three suspension samples of different dilution ratios. Inoculate the original bacterial suspension and the bacterial suspensions of different dilution ratios separately into the PDA medium added with chloramphenicol. For each dilution ratio, two petri dishes are provided for inoculation, and 0.5 mL of bacterial suspension is inoculated in each Petri dish. After inoculation in each Petri dish is completed, shake the petri dish to ensure full mixing, and put the petri dish into the constant temperature and humidity incubator for cultivation at 28 °C for 48 h. After that, take out the petri dish and calculate the *TVC* value of the rice grain and perform regression analysis on the *TVC* and *MAI* values.

## 3. Results

### 3.1. Variation of Box Loss during Model Training

**Figure 6:**
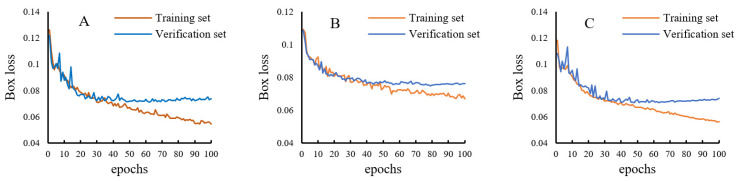
Variation of box loss during model training. (**A**) *A. niger*; (**B**) *P. citrinum*; (**C**) *A. cinerea*.

It can be seen from Figure 6 that during the process of establishing the three mildewed region recognition models, the variation of box loss is consistent. At the initial stage of training, the box losses of the training set and the prediction set decrease rapidly. After 30 epochs, the box loss of the verification set tends to be stable, while the box loss of the training set continues to decline, indicating that the model has started to over-fit. Therefore, the best identification model can be obtained after about 30 training epochs, and the box loss is around 0.08. After 30 epochs, an excessive number of training epochs will lead to over-fitting of the model.

### 3.2. Accuracy of Mildewed Region Detection Model

**Figure 7:**
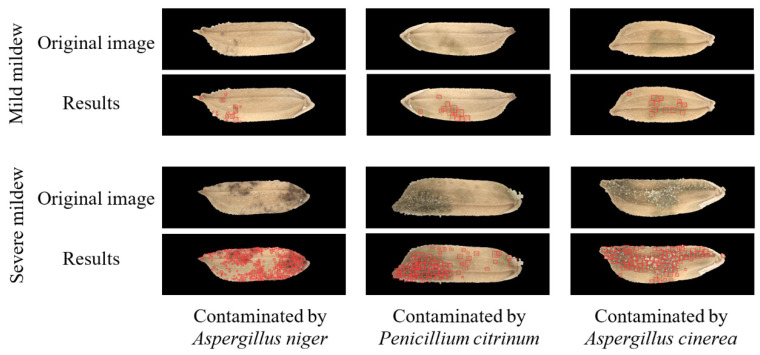
The effectiveness of the proposed model in detecting mildewed regions in microscopic images of rice grains contaminated by three mold strains.

Table 1 shows the confusion matrices of the recognition results yielded by the three YOLO-v5 models in detecting mildewed regions in the microscopic images of rice grains contaminated by the corresponding mold strains. For the training and verification sets of the microscopic images of rice grains contaminated by *A. niger*, the recognition accuracies of mildewed regions (the number of correctly identified pixels in the marked mildewed regions/total number of pixels in the marked mildewed regions) are 90.23% and 89.26%, respectively. For the training and verification sets of the microscopic images of rice grains contaminated by *P. citrinum*, the recognition accuracies of mildewed regions are 91.45% and 91.15%, respectively. For the training and verification sets of the microscopic images of rice grains contaminated by *A. cinerea*, the recognition accuracies of mildewed regions are 91.16% and 90.19%, respectively. For the training and verification sets of the microscopic images of rice grains contaminated by *A. niger*, the recognition accuracies of the background area (the number of correctly identified pixels in the background area/total number of pixels in the background area) are 96.10% and 95.72%, respectively. For the training and verification sets of the microscopic images of rice grains contaminated by *P. citrinum*, the recognition accuracies of background area are 94.16% and 93.96%, respectively. For the training and verification sets of the microscopic images of rice grains contaminated by *A. cinerea*, the recognition accuracies of the background area are 91.20% and 91.93%, respectively. Figure 7 shows the microscopic images of two groups of rice grains contaminated by molds before and after model recognition. The grains of one group have mild mildew (*TVC* is in the range of 10^5^–10^6^ CFU/g), and the grains of the other group have severe mildew (*TVC* > 10^6^ CFU/g). It can be seen from the figure that the mildewed region in the image can be effectively recognized by the model without being affected by other information in the image. Moreover, the recognition speed of YOLO-v5 models is very fast. After timing, the recognition time of one single image is about 0.04 s.

### 3.3. Analysis of Feature Images in the Middle Layer of the Model

**Figure 8:**
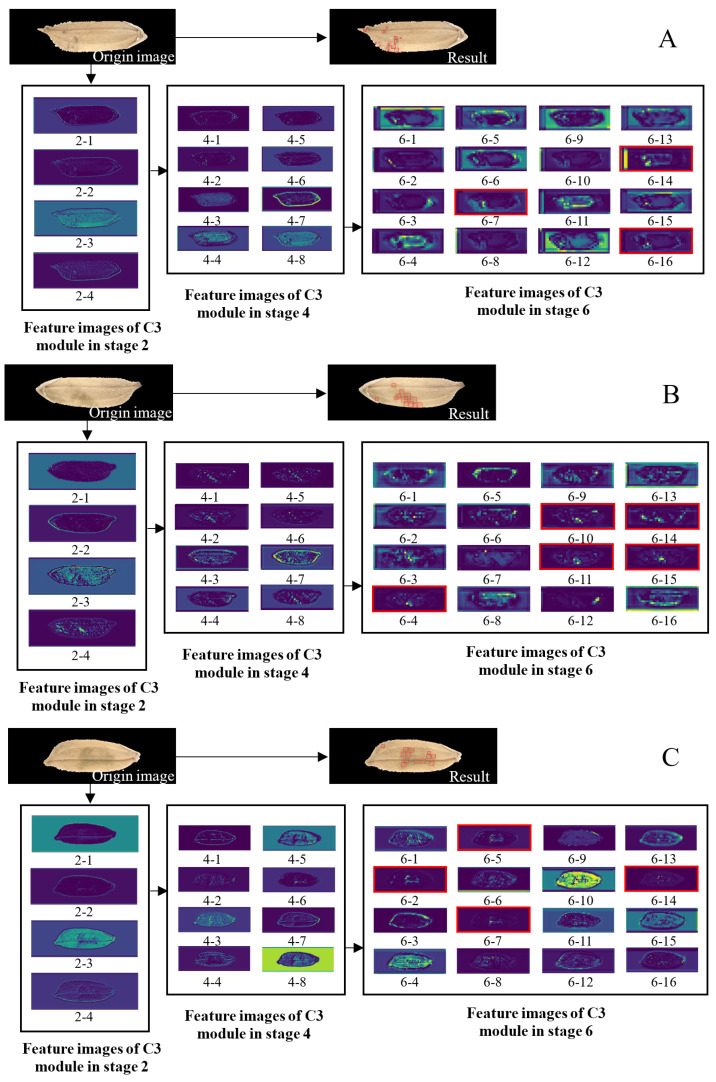
Some feature images output by the C3 module of the backbone of YOLO-v5. (**A**) *A. niger*; (**B**) *P. citrinum*; (**C**) *A. cinerea.* Feature images of C3, in which the mildewed region is well extracted, are highlighted by red boxes.

As shown in Figure 5 and Figure 8, the feature images of the C3 module in stage 2 of the model are obtained by convolution and feature extraction and fusion (performed on the original images). There are 64 feature images (the first four images are shown in Figure 8), with a resolution of 320 × 80 pixels. The feature images of the C3 module in stage 4 are obtained by convolution (performed on the feature images of the C3 module in stage 2) and feature extraction and fusion. There are 128 feature images (eight images are shown in Figure 8), with a resolution of 160 × 40 pixels. The feature images of the C3 module in stage 6 are obtained by convolution (performed on the feature images of the C3 module in stage 4) and feature extraction and fusion. There are 256 feature images (16 images are shown in Figure 8), with a resolution of 80 × 20 pixels. The feature images of the deeper network modules have very low resolution and poor readability, so they are not shown here.

The feature images of the C3 module in stage 2 indicate that the whole rice grain, grain edge, high-frequency information of the grain, and information on mildewed regions can be obtained after the model executes the C3 module in stage 2. However, the information about mildewed regions cannot be extracted separately and completely. The feature images of the C3 module in stage 4 indicate that more information about mildewed regions can be extracted after the model executes the C3 module in stage 4, and there is significantly less interference information. The feature images of the C3 module in stage 6 indicate that the interference during the process of mildew information extraction diminishes after the model executes the C3 module in stage 6. For some images, the extracted features of mildewed regions already fit very well with the final recognition results. In summary, after the execution of the C3 module in stage 6, the features of the mildewed regions in the rice grain image are obtained.

### 3.4. Analysis of Relationship between TVC and MAI of Rice Grain

**Figure 9:**
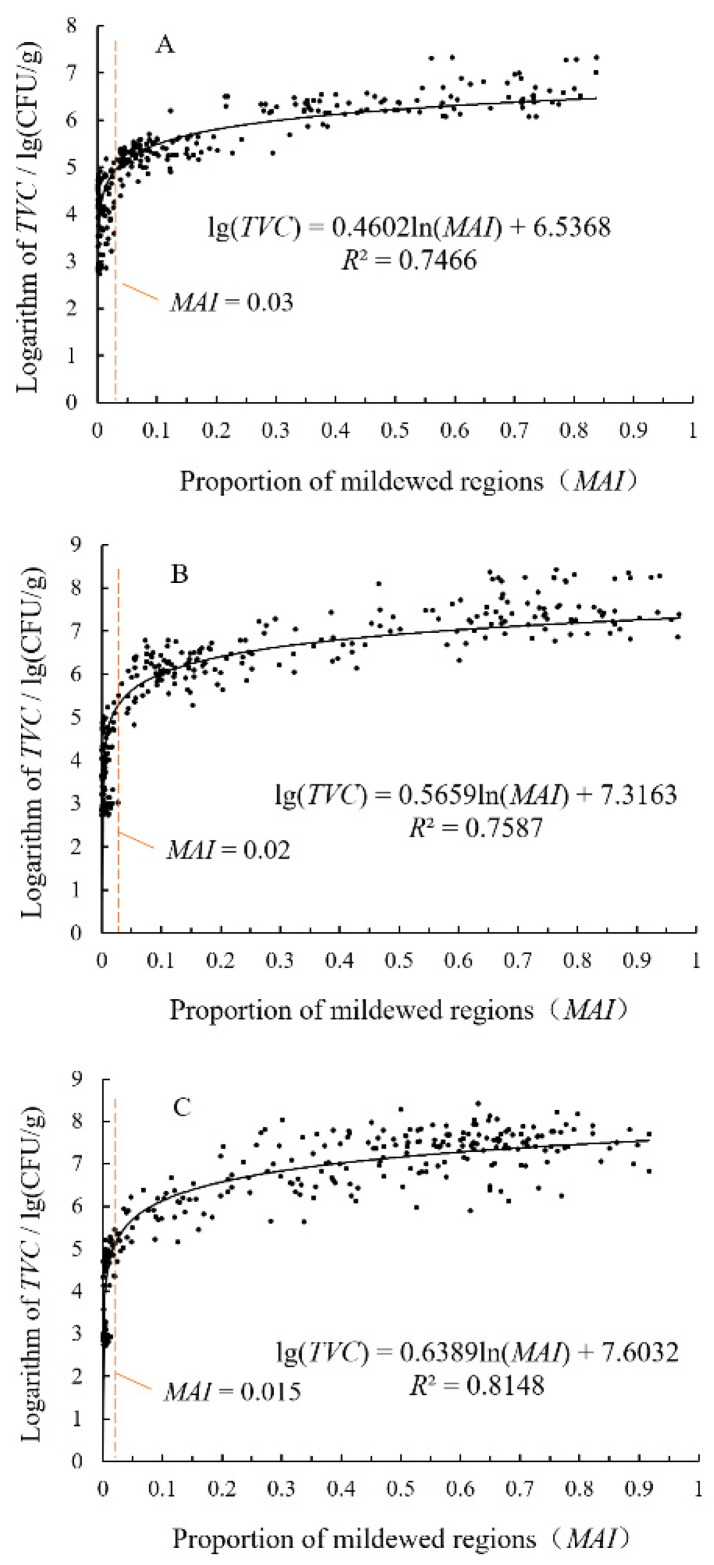
The relationship between *TVC* and *MAI* of rice grains contaminated by different molds. (**A**) *A. niger*; (**B**) *P. citrinum*; (**C**) *A. cinerea*.

Figure 9 shows the relationship between *TVC* and *MAI* of rice grains contaminated by *A. niger*, *P. citrinum*, and *A. cinerea*. The *MAI* of rice grains contaminated by *A. niger*, *P. citrinum*, and *A. cinerea* is logarithmically correlated with the logarithm of the *TVC* of the grains. The corresponding determination coefficients of the regression model are 0.7466, 0.7587, and 0.8148, respectively. Specifically, 110 grains among 121 rice grains with an extremely low level (*TVC* < 10^5^ CFU/g) of *A. niger* contamination have *MAI* values lower than 0.03 (90.9%), and 90 grains among the 93 rice grains with a mild (10^5^ ≤ *TVC* < 10^6^ CFU/g) *A. niger* contamination have *MAI* values greater than 0.03 (96.8%). Similarly, 96 grains among the 99 rice grains with an extremely low level of *P. citrinum* contamination have *MAI* values lower than 0.02 (96.9%), and 31 grains among the 33 rice grains with mild *P. citrinum* contamination have *MAI* values greater than 0.02 (93.9%). Lastly, 70 grains among the 75 rice grains with an extremely low level of *A. cinerea* contamination have *MAI* values lower than 0.015 (93.3%), and 31 grains among the 36 rice grains with mild *A. cinerea* contamination have *MAI* values greater than 0.015 (86.1%). It can be seen that there is a clear threshold value that distinguishes the *MAI* values of the rice grains with an extremely low mildew degree from those of the rice grains with a mild mildew degree. However, the threshold value changes with the change in the mold species. On the contrary, there is no clear threshold value to distinguish the *MAI* values of the rice grains with a high mildew degree (*TVC* > 10^6^ CFU/g) from those of the rice grains with a mild mildew degree. There is only a weak correlation between the logarithm of the *TVC* value of the rice grains with high mildew degree and the *MAI* values of those grains.

## 4. Discussion

Until now, not much research has been conducted to explore the methods for testing the quality of grain-based food products based on the MCV technology. Bhupendra et al. (2022) acquired some images of various rice grains using a camera with 4.5× optical magnification lens and established a model for grading rice grain impairments [31]. In this study, a camera with 2× optical magnification lens was used for image acquisition. For digital microscopic images, the resolution of the camera used to acquire images also has a great impact on the spatial resolution of the final image. The resolution of the camera used in this study is 4096 × 3000, and the spatial resolution is 0.01 mm/pixel when the object is a single rice grain. Such resolution meets the requirements of image recognition. If the magnification continues to increase, the field of view of each image is reduced, which seriously affects the practicability of this method.

In most studies in which the YOLO is used to establish the target recognition model, the researchers consider the box loss and object loss of the model as well as the recall rate and average precision calculated from the former two indicators when evaluating the accuracy of the model [25,32]. In this study, the YOLO-v5 model is trained based on reducing the box loss without considering the object loss of the model. This is because mildewed regions on rice grain images usually have fuzzy and irregular boundaries, and obvious and isolated colonies are rare. In order to get the bounding box as close to the outline of the real mildewed region as much as possible in the process of image marking, we adopt a method that uses multiple small boxes (smaller than 200 × 200 pixels) for marking due to the infeasibility of using larger marking boxes. As a result, the marking boxes only contain texture features formed by mildew and lack obvious morphological features. This means different marking boxes do not represent independent mildewed regions, thus making it impossible to achieve convergence of object loss during model training. In contrast, in the study of the defect detection for bulk grain and soybean [33,34], lower object loss can be obtained using the CNN model because the objects segmented are intact bean grains, the shape of recognition targets is close to a circle, and the boundary delineation is clear. With an increase in the number of training epochs, the box loss of the model continuously decreases, and the degree of coincidence between the mildewed region resulting from the superposition of the output marker boxes and the manually marked mildewed region continues to improve. This indicates that the failure of target loss convergence does not affect the model’s performance in identifying mildewed regions of rice grains. The role of the YOLO model in this study is similar to image segmentation, i.e., divide the image into mildewed regions and background areas. Therefore, we use the confusion matrix of the recognition results yielded by the model in detecting mildewed regions and background areas in rice grain images to evaluate the accuracy of the model.

As shown by the *MAI* values of the rice grain samples calculated using the proposed mildewed region detection model, rice grains with an extremely low level of contamination of *A. niger*, *P. citrinum*, and *A. cinerea*, and the rice grains with mild contamination of the three mold strains can be accurately distinguished using *MAI*. However, the threshold value of *MAI* for distinguishing mildew degrees changes with the change in mold strain, which may be attributed to the differences between mold strains in diffusion ability and visibility during the process of rice grain mildewing. Some rice grains with mild contamination of *A. cinerea* can be easily misjudged as grains with an extremely low mildew degree. This is because *A. cinerea* only exhibits itself in the form of tiny, light-colored hyphae in the early stages of proliferation on the surface of rice grains, making it difficult to detect this mold using a computer vision system.

Through this study, it was found that the established YOLO convolutional neural network model can effectively identify the mildewed regions on a single rice grain, and the proportion of the mildewed regions identified has the potential to discriminate mildly mildewed rice grains. However, the following studies need to be conducted before this method can be practically applied to mildewed rice grain detection: (1) this study only built the recognition model of the mildewed region of a single rice grain, and the recognition model of grouped rice sample needs to be established by transfer learning in the future; (2) this study only used three kinds of mold, but rice grains can be contaminated by other mold or multiple kinds of mold. Thus, more mildewed rice grain samples need to be collected to establish the recognition models; (3) Compared with conventional computer vision [10], microscopic image capture will inevitably lead to a reduced field of view for sample observation, and how to acquire representative microscopic images for grouped rice grain samples needs further study.

## 5. Conclusions

In this paper, we developed a YOLO-v5 convolutional neural network model for identifying mildewed regions in microscopic images of rice grain contaminated with *A. niger*, *Penicillium oryzae*, and *Aspergillus griseofulgens* and studied the detection effect of mildewed rice grain based on the CVT and YOLO-v5 models. The following conclusions are drawn.

(1) YOLO-v5 models for identifying mildewed regions in the microscopic images of rice grains contaminated by *A. niger*, *P. citrinum*, and *A. cinerea* are established. The proposed models achieve quite good results in an experiment performed using a set of prepared rice grain samples. The accuracies of identifying the mildewed regions with contamination of *A. niger*, *P. citrinum*, and *A. cinerea* in the microscopic images of the verification set are 89.26%, 91.15%, and 90.19%, respectively. (2) An accurate feature image of the mildewed region can be obtained after the model executes the C3 module in stage 6. (3) The *MAI* values of rice grains contaminated by *A. niger*, *P. citrinum*, and *A. cinerea* are logarithmically correlated with the logarithms of the *TVC* values of the grains. The corresponding determination coefficients are 0.7466, 0.7587, and 0.8148, respectively. The rice grains with an extremely low mildew degree and the grains with a mild mildew degree can be distinguished using an *MAI* threshold. The *MAI* threshold is 0.3 in the case of *A. niger* contamination, 0.2 in the case of *P. citrinum* contamination, and 0.15 in the case of *A. cinerea* contamination.

## Figures and Tables

**Table 1:** Confusion matrix of the mildewed region detection model for rice grains contaminated by three kinds of mold.

			Mildewed Region (×10^3^ Pixels)	Normal Region (×10^3^ Pixels)	Accuracy	Overall Accuracy
*A. niger*	Training set	Mildewed region	40,886	4426	90.23%	95.44%
		Background area	13,805	340,526	96.10%	
	Verification set	Mildewed region	29,599	3562	89.26%	94.93%
		Background area	10,195	228,245	95.72%	
*P. citrinum*	Training set	Mildewed region	64,657	6015	91.45%	93.73%
		Background area	21,477	346,273	94.16%	
	Verification set	Mildewed region	49,429	4801	91.15%	93.76%
		Background area	15,015	233,549	93.96%	
*A. cinerea*	Training set	Mildewed region	62,591	6071	91.16%	91.19%
		Background area	19,252	199,554	91.20%	
	Verification set	Mildewed region	40,747	4431	90.19%	91.52%
		Background area	11,556	131,715	91.93%	

## Data Availability

The data presented in this study are available on request from the corresponding author. The data are not publicly available due to the large size of image data, which make it difficult to upload.
